# MicroRNA-106b~25 cluster is upregulated in relapsed *MLL*-rearranged pediatric acute myeloid leukemia

**DOI:** 10.18632/oncotarget.10270

**Published:** 2016-06-24

**Authors:** Lonneke J. Verboon, Askar Obulkasim, Jasmijn D.E. de Rooij, Jenny E. Katsman, Edwin Sonneveld, André Baruchel, Jan Trka, Dirk Reinhardt, Rob Pieters, Jacqueline Cloos, Gertjan J.L. Kaspers, Jan-Henning Klusmann, Christian Michel Zwaan, Maarten Fornerod, Marry M. van den Heuvel-Eibrink

**Affiliations:** ^1^ Department of Pediatric Oncology, Erasmus MC-Sophia Children's Hospital Rotterdam, Rotterdam, The Netherlands; ^2^ Dutch Childhood Oncology Group (DCOG), The Hague, The Netherlands; ^3^ Department of Hematology, Hopital Saint- Louis, Paris, France; ^4^ Department of Pediatric Hematology/Oncology, 2nd Medical School, Charles University, Prague, Czech Republic; ^5^ Clinic for Pediatrics III, University Hospital Essen, Essen, Germany; ^6^ Princess Maxima Center for Pediatric Oncology, Utrecht, The Netherlands; ^7^ Paediatric Oncology/Haematology, VU University Medical Centre, Amsterdam, The Netherlands; ^8^ Department of Pediatric Hematology and Oncology, Hannover Medical School, Hannover, Germany

**Keywords:** acute myeloid leukemia, MLL, relapse, miR-106b~25

## Abstract

The most important reason for therapy failure in pediatric acute myeloid leukemia (AML) is relapse. In order to identify miRNAs that contribute to the clonal evolution towards relapse in pediatric AML, miRNA expression profiling of 127 *de novo* pediatric AML cases were used. In the diagnostic phase, no miRNA signatures could be identified that were predictive for relapse occurrence, in a large pediatric cohort, nor in a nested mixed lineage leukemia (*MLL)*-rearranged pediatric cohort. AML with *MLL*- rearrangements are found in 15-20% of all pediatric AML samples, and reveal a relapse rate up to 50% for certain translocation partner subgroups. Therefore, microRNA expression profiling of six paired initial diagnosis-relapse *MLL*-rearranged pediatric AML samples (test cohort) and additional eight paired initial diagnosis-relapse samples with *MLL*-rearrangements (validation cohort) was performed. A list of 53 differentially expressed miRNAs was identified of which the miR-106b~25 cluster, located in intron 13 of *MCM7*, was the most prominent. These differentially expressed miRNAs however could not predict a relapse in *de novo* AML samples with *MLL*-rearrangements at diagnosis. Furthermore, higher mRNA expression of both *MCM7* and its upstream regulator *E2F1* was found in relapse samples with *MLL*-rearrangements. In conclusion, we identified the miR-106b~25 cluster to be upregulated in relapse pediatric AML with *MLL*-rearrangements.

## INTRODUCTION

Acute myeloid leukemia (AML) is a heterogeneous disease characterized by various molecular and cytogenetic abnormalities, like chromosome rearrangements and mutations in different genes [1]. Although survival rates have improved over the last decade, still overall survival does not exceed 70% in most biological groups [2]. Pediatric AMLs with mixed lineage leukemia (*MLL)* rearrangements (or *KMT2A*, *lysine (K)-specific methyltransferase 2A*) represent about 20% of all pediatric AML patients [3]. The clinical outcome of this group, in general, is intermediate, however substantial differences in overall survival related to certain *MLL* translocation partners have been reported. In particular cases with t(10;11)(p12;q23), t(10;11)(p11.2,q23), or t(6;11)(q27;q23) represent a very poor-risk group, mainly due to early relapses. Approximately 50% of AML patients with t(10;11) and t(6;11) *MLL*-rearrangements will relapse during or after therapy [4]. The biological processes that determine relapse and subsequent therapy failure in *MLL*-rearranged AML are largely unknown.

Gene expression is regulated via transcription, RNA processing, and mRNA translation. Recently microRNAs (miRNAs) have been identified to control expression of relevant leukemogenic drivers. MiRNAs are the epigenetic fine-tuners of gene expression, and deregulation has been described in a variety of human cancer types, including adult AML [5]. MicroRNA genes are often found in chromosomal regions that are deleted, amplified or involved in translocations in cancer [6, 7]. MiRNAs can act as tumor suppressors as well as oncogenes [8]. This is determined by cell type and physiological context [9]. For instance, the oncogenic miR-17~92 cluster has been shown to be overexpressed in *MLL*-rearranged acute leukemias probably due to amplification of the genomic locus 13q31.3 and by direct upregulation caused by *MLL* fusion genes [10, 11]. Due to epigenetic modifying properties, miRNAs play an important role in the translation of mRNA into protein [12].

However, to date, the role of miRNAs in leukemogenesis in pediatric AML, especially in the process of clonal expansion towards relapse is limited. The aim of this study was to determine whether specific miRNAs are involved in, or can predict relapse development in general and, in particular, in *MLL*-rearranged pediatric AML.

## RESULTS

MicroRNA profiling in a cohort of 127 *de novo* AML cases was performed recently by Emmrich *et al* [13]. To explore whether relapse can be predicted in *de novo* setting of pediatric AML, we compared miRNA expression profiles of *de novo* pediatric AML patients that eventually relapsed, with those that did not relapse. Subsequently, we performed the same comparison, only in the nested subset *MLL*-rearranged AML cases. Of 127 *de novo* AML cases, 59/127 (46.5%) relapsed after complete remission (S1 Table). No statistically significant miRNAs were identified when we compared relapsing and non-relapsing cases at diagnosis (*p*=0.643), neither when type II aberrations were used as a confounder. Also in the nested cohort of *de novo* pediatric AML *MLL*-rearranged cases (4 *AF6*, 7 *AF9*, 9 *AF10*, 1 *FNBP1*, 1 *SEPT6*, and 1 *NRIP3*)(S2 Table), of which 14/23 (60.9%) relapsed, no difference in miRNA expression profile was found between cases that relapsed and cases that recurred (*p*=0.429).

### MiRNAs are differentially expressed in initial diagnosis-relapse samples with *MLL*-rearrangements

**Figure 1:**
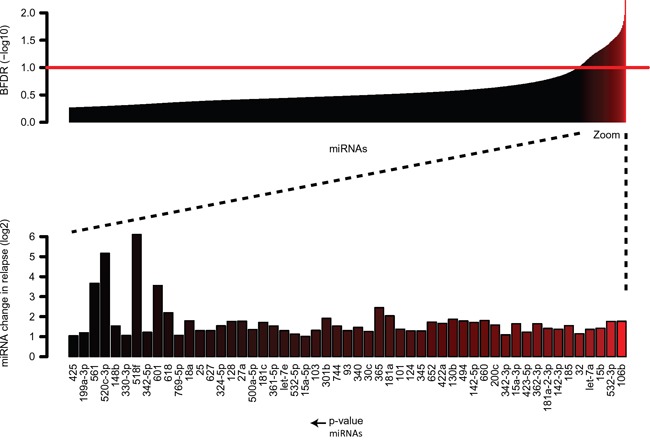
MicroRNA expression in paired initial diagnosis-relapse samples with *MLL*-rearrangements as determined by Taqman Low Density Arrays Expression of 53 differentially expressed miRNAs as measured by TLDA of 6 paired pediatric AML initial-relapse cases with *MLL*-rearrangements. Expression is significantly higher in relapse samples as compared to initial diagnosis. Data are presented as median miRNA changes in relapse (BFDR<0,1).

To investigate a potential role for miRNAs in clonal evolution towards relapsed disease in pediatric *MLL*-rearranged AML, we conducted miRNA expression profiling in a cohort of six paired initial diagnosis-relapse samples with various *MLL*-rearrangements (Table 1). A total of 53 significantly (BFDR<0.1) differentially expressed miRNAs were identified using the Bayesian approach (Figure 1). We observed that all these miRNAs were overexpressed (≥2 fold change) in the relapse samples compared to the initial diagnosis. None of the 53 miRNAs, turned out to be significant when were used to discriminate the *de novo* pediatric *MLL*-rearranged AML cases that relapsed from those that did not relapse based on miRNA profiles at diagnosis (*p*= 0.193).

**Table 1:** Characteristics of pediatric *MLL*-rearranged AML patients included in the paired sample study

ID	Array	Age (y)	Sex	WBC (x 10^9^/L)	FAB	Karyotype sample	TP	Type 1 Diagnosis	Type 1 Relapse
1	MC	1.6	M	16.1	M5	Dx: 46, XY,del(10)(p12), der(11)(t(10;11)(p12;q2?3)[20] R: N/A	AF10	NRAS	None
2	MC	0.3	F	NA	NA	Dx: Unknown R: Unknown	AF9	None	None
3	MC	1.2	M	23.0	M7	Dx: 46, XY, t(9;11)(p22;q23) R: 46, XY [12], 47, XY, t(9;11)(p22;q23), ?del(18)(q2?1), +19 [1], 50~53, XY, +6, +6[4], del(9)(q3?3), +del(9)(q3?3)[5], t(9;11)(p22;q23), −18, ?de;(18)(q2?1), +19, +20, +21, +21, +mar1, +mar1 / 46, XY	AF9	None	None
4	MC	12.8	M	2.5	M5	Dx: 46, XY R: 46, XY, ?del(11)(p15)[cp5]	AF10	None	None
5^†^	MC	1.9	M	237.0	M5	Dx: 46, XY, add(11)(q23)[19]/46,XY[2] R: 46, XY, add(11)(q23)[24]	AF10	None	KRAS
6^†^	MC	7.6	M	129.0	M5	Dx: 46, XY, ?t(3;11)(q26;q12) inv(11)(q12q23)]/46, XY[1] R: 53,XY,?t(3;11)(q26;q21),+6,+8,+18,+19,+21,+21,+22 ish t(3;11)(q26;q12)inv(11)(q12q23) [10]/ 46,XY [6]	AF10	NRAS	None
7^†^	V	5.3	M	34.0	M4 or M5	Dx: 46,XY,add(11)(q23),del(12)(p11p13)[4]/46,XY[22] R: 46, XY[20]/add(11q23)del(12)(p12-13)[4]	Unk	None	None
8	V	1.4	F	3.2	M5	Dx: 46,XX,der(10)ins(10;11)(p12;q23q13),der(11)?der(11)(p1?)ins(10;11) [13]/ 46,XX [9] R: 46,XX,der(10)ins(10;11)(p12;q23q13),der(11)?der(11)(p1?)ins(10;11) [13]/ 46,XX [9]	AF10	None	None
9	V	10.8	M	67.0	M5	Dx: 46,XY[20] R: 46,XY[20] ish ins (10;11)(p;q23q23) (5′ MLL+) [6/10]	AF10	NRAS	None
10	V	9.5	F	45.7	M0	Dx: 51~53,XX,+2,+4,+6,+10,+add(11)(p?15),+13,−18,+21,+22,inc[cp10] R: ND	AF10	FLT3-ITD^1^	None
11	V	14.1	M	42.0	M5	Dx: 46~47,X,der(Y)t(Y,1)(q12,q12),der(8;12)(q10,q10),+1-2mar [8] R: 46~47,X,der(Y)t(Y,1)(q12,q12),der(8;12)(q10,q10),+1-2mar [8]	AF10	None	None
12	V	8.5	M	106.0	M1	Dx: 46,XY,add(11)(q23),inc R: 46,XY,add(11)(q23),inc	ELL	None*	WT1
13	V	10.6	F	5.5	M5	Dx: 47, XX, +8, cryptic ins(10;11)(p1?;q23q23) R: 45~50, XX, der(1)add(1)(p36)add(1)(q2?1), der(1)add	AF10	NRAS	None
14	V	11.4	M	ND	M5	Dx: 47,XY,+8,t(11;19)(q23;p13.3)[9]/46,XY[1] R: 47,XY,+8,t(11;19)(q23;p13.3)[6]/46,XY[4]	ENL	None	None

Abbreviations: WBC indicates white blood cell count; FAB, French American British morphology classification; ND, not determined; NA, not available; Dx, diagnosis; R, relapse; Type I mutations screened for NRAS, KRAS, FLT3, PTPN11, KIT and WT1. ^1^) heterozygote;

* not screened for WT1;

† used for Western blot. MLL translocations were determined either with FISH or RT-PCR

To confirm these findings, we selected 22 miRNAs, the top ten most differentially expressed miRNAs (Supplementary Figure S1) and 12 potential oncogenic miRNAs from literature, out of 53 significant miRNAs for validation with stem-loop RT-qPCR. A replication set of eight additional initial diagnosis-relapse samples with *MLL*-rearrangements was included in this analysis (Table 1). Among these 22 miRNAs, 12 were confirmed to be significantly upregulated in the relapse samples (Wilcoxon signed rank test; *p*<0.05), nine failed to pass the significance threshold, and one miRNA could not be detected in any samples.

**Figure 2:**
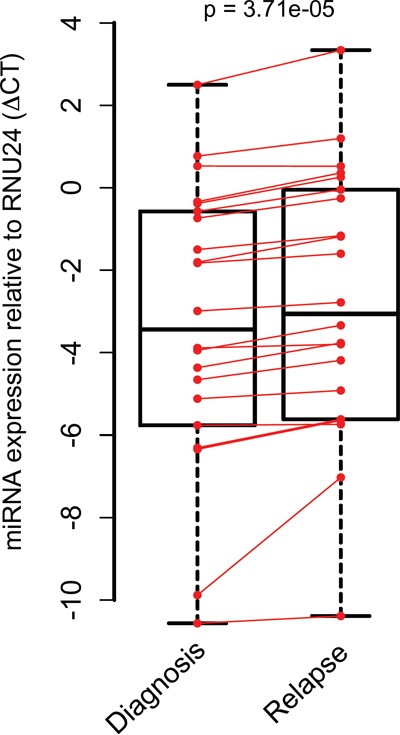
MiRNA expression in paired pediatric AML initial diagnosis-relapse cases with stem-loop RT-qPCR Median miRNA expression levels were determined with single stem loop RT-qPCR per miRNA between initial diagnosis and relapse and are significantly different. A large spread is found in miRNA expression levels of validated miRNAs. Data is presented with one-sided P-value.

The one-sided Wilcoxon signed-rank test was used to assess differential expression of the 22 selected miRNAs between initial and relapse samples. In subsequent analyses, miRNA-532-3p was excluded as it was undetectable with RT-qPCR. A significant overall difference was found in miRNA expression between the initial and relapse (global test *p*<0,001)(Figure 2 and Supplementary Table S3).

### MiR-106b-25 cluster is overexpressed in relapsed *MLL*-rearranged AML

**Figure 3:**
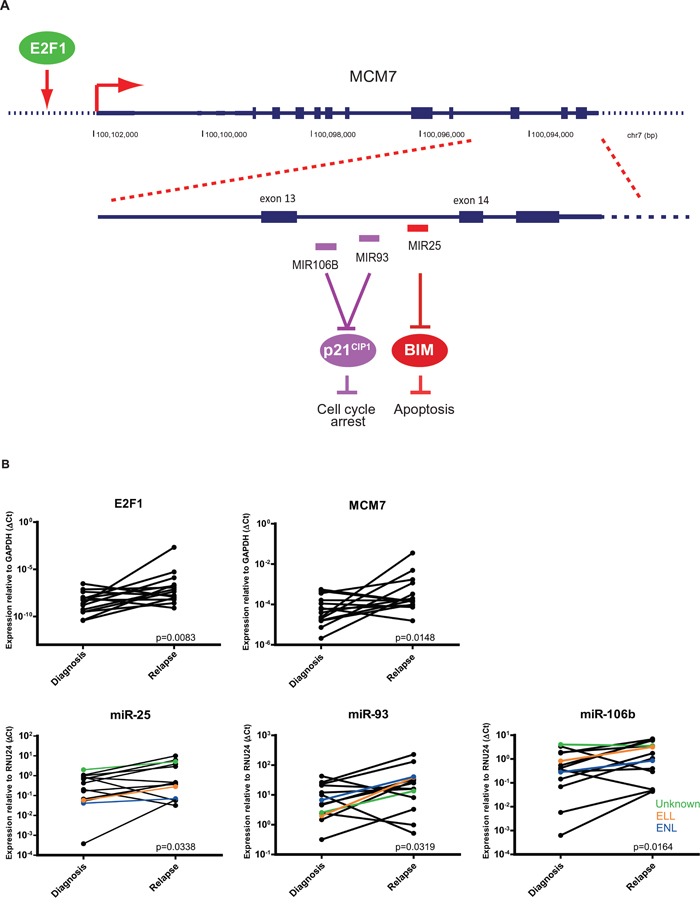
Relative expression of *E2F1*, *MCM7*, and miR-106b~25 cluster **A.** The miR-106b~25 cluster in located intron 13-14 of MCM7 and cotranscribed together as part of the MCM7 primery RNA transcript. E2F1 regulates MCM7 expression. **B.** Relative miRNA expression of miR-25, miR-93, and miR-106b in 14 paired initial diagnosis-relapse pediatric AML samples with *MLL*-rearrangements and mRNA expression of *E2F1* and *MCM7*. Patients without AF9 or AF10 translocation have comparable miR-106b~25 expression (colored lines). Data are presented with one-sided p-values.

Among the 53 identified overexpressed miRNAs, miR-106b-5p was ranked as one of the most prominently overexpressed miRNAs. MiR-106b clusters together with miR-93-5p and miR-25-3p (miR-106b~25 cluster) in intron 13 of minichromosome maintenance complex component 7 (*MCM7*) on chromosome 7, which were part of the top list differentially expressed miRNAs. This cluster is actively co-transcribed as part of the *MCM7* primary RNA transcript [14]. E2F1 transcription factor 1 (*E2F1*) acts as an upstream regulator of MCM7 (Figure 3A). *MCM7* mRNA overexpression has previously been shown to be associated with poor outcome in solid tumors. Potentially, this may be regulated by overexpression of the hosted miRNAs [15, 16].

Therefore, we studied *MCM7* and *E2F1* mRNA (RT-qPCR) expression and could show that they were differentially overexpressed in the paired samples with *MLL*-rearrangements at relapse (n=14, *p*=0.008 and *p*=0.015, respectively)(Figure 3B). Overexpression of miR-106b, miR-93 and miR-25 at relapse by miRNA profiling, was confirmed by stem loop RT-qPCR in the paired patient samples, both in the replication cohort (n=8) as in the discovery cohort (n=6) (*p*<0.05)(Figure 3B).

The expression of the three miRNAs in the miR106b~25 cluster were, in general, consistently up- or downregulated. Among the 14 cases, four patients had one miRNA that showed opposite expression compared to the other two miRNAs within the cluster (Supplementary Figure S2). It is conceivable, that post-translational modifications such as alternative splicing of the miR-106b~25 transcript may occur, thereby separately regulating miR-25 [17], and mutations in the miRNA transcript may have induced this difference in expression. Therefore, we screened intron 13-14 of MCM7 for mutations. We did not identify mutations in intron 13-14 of MCM7 and observed that the frequency of the SNP rs1527423 was similar to that in the normal human population (data not shown). All samples (either AF9, AF10 or other TPs) showed similar miRNA expression (Figure 3B).

### Downstream targets of the miR-106b~25 cluster

**Figure 4:**
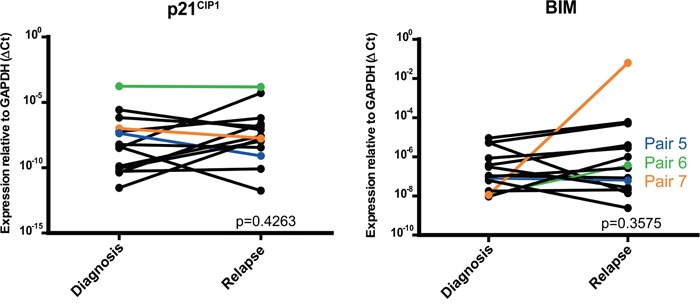
Relative expression of *p21*WAF1/*CIP1 and BIM* in paired pediatric AML initial diagnosis-relapse cases with RT-qPCR (Taqman) Patients used for Western blot have a colored line. Dara are presented with one-sided p-values

Multiple downstream targets for the miR-106b~25 cluster have been described in different cancer types. We selected two predicted targets of the miR-106b~25 cluster, cyclin-dependent kinase inhibitor 1A (*p21^WAF1/CIP1^*) and BCL2-like 11 (*BIM*) [18], based on protein function and relevance of these proteins in cancer. In the 14 paired samples we did not find differences in *p21^WAF1/CIP1^* (*p*=0.43) or *BIM* (*p*=0.36) mRNA expression between initial diagnosis and relapse (Figure 4). Of the five paired initial diagnosis-relapse samples with *MLL*-rearrangements gene expression profiles (GEP) were available (2 *AF9* and 3 *AF10*). Although 22 genes were found to be negatively correlated with the 53 differentially overexpressed miRNAs, *p21^WAF1/CIP1^* and BIM were not negatively correlated with GEP (data not shown).

**Figure 5:**
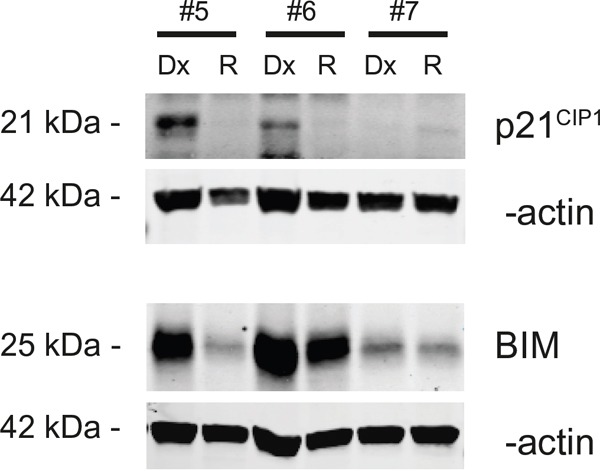
Protein expression anaylis of p21WAF1/CIP1 and BIM with Western blot Three patients of which protein was available were used to validate protein expression of p21^WAF1/CIP1^ and BIM. Patients 5 and 6 have a MLL-AF10 rearrangement.

MiRNAs regulate translation of genes through translational repression or target mRNA cleavage. Although no difference in mRNA expression of *p21^WAF1/CIP1^* and *BIM* could be found, we anticipated that protein level could be different. For that reason, we used western blot analysis on three paired initial diagnosis-relapse samples with *MLL*-rearrangements (Table 1). Among two out of three paired initial diagnosis-relapse samples, the expression of *BIM* and *p21^WAF1/CIP1^* was downregulated in relapse samples as compared to that of initial diagnosis, both in patients with a MLL-AF10 translocation. One patient showed a modest downregulation of *BIM* and almost no *p21^WAF1/CIP1^* protein expression (Figure 5).

As both *p21^WAF1/CIP1^* and *BIM* are validated targets in prostate cancer and human mammary epithelial cells [18, 19], we studied p21^WAF1/CIP1^ and BIM protein expression in two *MLL*-rearranged cell lines, Nomo1 and THP-1, after overexpression of the miR-106b~25 cluster. However, we found no decreased in BIM protein expression upon forced expression of this cluster, while neither *MLL*-rearranged cell line had detectable p21^WAF1/CIP1^ expression (data not shown). Therefore, the identity of these two genes as miR-106b~25 target remains hypothetical in our study.

## DISCUSSION

Over the last years, miRNAs are widely studied in the context of cancer development, progression, and cancer type classification [20]. Based on miRNA profiling, AML samples could be classified according to FAB type [21] or cytogenetic information [13]. However, the contribution of miRNAs in the process of clonal expansion towards relapse in pediatric AML has not been reported so far. In this study we aimed to investigate the potential role of miRNAs in clonal evolution towards relapse by miRNA expression profiling of paired initial diagnosis-relapse samples with *MLL*-rearrangements.

In our series of pediatric AML we could not identify miRNAs that were predictive for relapse occurrence in pediatric AML cases in the diagnostic phase. This may have been due to the heterogeneity of selected cases (n=127). However, also in a nested more homogeneous cohort of *MLL*-rearranged AML cases, we did not identify a miRNA signature that could predict relapse. We appreciated the fact that although we studied 23 cases (of which 9 carried a MLL-AF10 translocation) this may, in part, have been due to insufficient power to find a specific miRNA signature predictive for relapse in poor risk *MLL*-AML cases. We identified 53 miRNAs differentially expressed miRNAs in the development towards relapse in *MLL*-rearranged pediatric AML. These differentially expressed miRNAs could however again, not predict relapse. Also, this may again underscore the fact that clones that are leukemia drivers at relapse, may exist only on the subclonal level as has been revealed by NGS studies recently [22, 23].

Our results suggest that alternative miRNAs are involved in the development of a relapse. This is in line with recent findings of Bachas *et al.* that revealed that alternative gene expression profiles and biological pathways, are relevant for relapse development, in a paired sample analyses of relapsed pediatric AML. Approximately, 48% of these paired samples did not culminate in the same gene expression cluster. Also, paired samples that were in the same cluster had differentially expressed genes [24]. This may underscore our findings that relapse reflects clonal evolution of small undiscovered clones that may be regulated by epigenetic mechanisms, rather than by individual genes.

Of the 22 miRNAs that were validated with stem-loop RT-qPCR, 12 were significantly upregulated, including three members of the miR-106b~25 cluster. This cluster has also been identified as an oncogenic microRNA cluster in other types of cancer, such as gastric, prostate, and breast cancer [15, 25, 26]. In total, 19 (out of 22) miRNAs that were detectable are more highly expressed at relapse compared to initial diagnosis as predicted in the discovery cohort. Validation of miRNAs obtained by TLDA arrays, especially in small cohorts, is required to determine the true significantly different miRNAs.

In four patients, one miRNA within the miR-106b~25 cluster showed opposite expression. This difference could not be explained by the presence of SNP rs1527423. No other mutations were found in the miRNA gene. Although single miRNAs from this cluster were reported in adult AML. Higher miR-25 expression was found to be correlated with adverse survival [27], and higher miR-93 serum levels were described to be valued as potential biomarker for detecting AML in adults [28].

In addition, in the study performed by Emmrich *et al* [13] only miR-106b was significantly higher expressed in pediatric AML subtype t(15;17)(q22;q21) (PML/RARA). Likewise, only miR-25-5p was significantly lower expressed in t(15;17). These findings may underline the fact that there is a particular role for this cluster in AML leukemogenesis.

We found overexpression of miR-106b~25, E2F1, and MCM7 at relapse. The miR-106b~25 cluster, located in intron 13 of the *MCM7* gene, is actively co-transcribed during transcription. It is conceivable that overexpression of the miR-106b~25 cluster is regulated by overexpression of *MCM7*. MCM7 is essential for the initiation of eukaryotic genome replication and ensuring that the whole genome is replicated once per cell cycle [29]. Expression of MCM7 is regulated via the activation of the transcription factor E2F1 which controls the expression of genes encoding various DNA replication proteins and cell cycle regulators [30]. Overexpression of E2F1 may lead to inappropriate DNA replication and subsequent formation of DNA double-stranded breaks result in DNA damage [31], thereby resulting in deregulation of hematopoiesis.

Two predicted downstream targets that are targeted by the miR-106b~25 cluster were further studied in depth, *p21^WAF1/CIP1^* and *BIM. W*e could not show deregulated mRNA expression of *p21*^WAF1/*CIP1*^ and *BIM* at relapse in the total group of *MLL* rearranged patients. However, 3 patients (1 AF9, 1 AF10, and 1 ELL) had both lower mRNA expression of *p21*^WAF1/*CIP1*^ *and BIM* at relapse and 2 additional patients (2 AF10) had lower *p21*^WAF1/*CIP1*^ expression at relapse. Difference in protein expression could be shown in the three paired initial diagnosis-relapse samples. Two of the three pairs showed lower p21^WAF1/*CIP1*^ and BIM protein expression in relapse of paired initial diagnosis-relapse samples (AF10). Previous studies showed, that, not only miR-106b~25 cluster targets p21^WAF1/*CIP1*^ and BIM, but also miR-30c and miR-32 have BIM as validated target [32, 33]. We could show that miR-30c and miR-32 were also overexpressed at relapse (Supplementary Figure S1). In addition hsa-let-7a, hsa-let-7e, and miR-301b, predicted by targetscan and miRanda to target p21^WAF1/*CIP1*^ could also contribute to the downregulation of these two targets.

In conclusion, we show that miRNA signatures could not predict relapse in a representative cohort of pediatric AML, nor in a nested series with *MLL*-rearrangements. Based on paired initial diagnosis-relapse miRNA analysis we identified 53 miRNAs that may be involved in clonal evolution of in the paired samples in pediatric *MLL*-rearranged AML. The miR-106b~25 cluster may be involved in the development of relapse of *MLL*-rearranged AML which may be caused through upregulation of *E2F1*. Downstream predicted targets of this cluster such as p21^WAF1/*CIP1*^ and BIM, may be repressed.

## MATERIALS AND METHODS

### Patient samples

Viably frozen bone marrow or peripheral blood samples from 14 paired initial diagnostic and relapsed (n=28) pediatric AML cases were provided by the Dutch Childhood Oncology Group (DCOG), the AML ‘Berlin–Frankfurt–Münster’ Study Group (AML-BFM-SG), the Czech Pediatric Hematology Group (CPH) and the St. Louis Hospital in Paris, France. Informed consent was obtained from all patients, after Institutional Review Board approval, according to national law and regulations. After thawing, samples were enriched to contain at least 80% leukemic blast cells as previously described [34]. Total cellular RNA and genomic DNA were extracted using TRIzol reagent (Invitrogen Life Technology, Breda, The Netherlands), as previously described [35].

### MiRNA and mRNA expression profiling and RT-qPCR

MiRNA expression profiling of leukemic cells from six paired initial diagnosis-relapse samples AML cases were performed. MiRNA expression was measured on Taqman Low Density Arrays (TLDA) v2.0 using Taqman technology in the 7900 HT Real-Time PCR System (Applied Biosystems, Foster City, CA, USA) according to the manufacturer's instructions.

Differentially expressed miRNAs were validated by single stem loop real-time PCR using TaqMan MiRNA assay (Applied Biosystem) according to the manufacturers' protocol. The expression levels of miRNA target genes (*BIM* and *p21^WAF1/CIP^*) that had been identified in other types of cancer, were validated with RT-qPCR in 14 paired initial diagnostic-relapse *MLL*-rearranged AML samples, six pairs in the discovery cohort and eight additional paired samples with *MLL*-rearrangements (9 AF10, 2 AF9, 1 ELL, 1 ENL, and 1 unknown) in the validation cohort. Primers are shown in Supplementary Table S4. Messenger-RNA expression was performed by RT-qPCR using DyNAmo HS SYBR green (Thermo Scientific, Waltham, MA, USA). The average cycle threshold (Ct) value was used to calculate miRNA and mRNA expression levels relative to the expression level of the reference RNU24 or *GAPDH*, respectively, using the comparative Ct method [36].

### Western blot

For Western blot, leukemic cells of three paired initial diagnosis-relapse samples of patients with *MLL*-rearrangements (2 AF10 and 1 unknown) were selected. Cells were lysed with Kinase Lysis buffer (KLB) [37], 25 μg protein per lane was loaded on precast polyacrylamide gel (10%, BIO-RAD, Hercules, CA, USA), transferred to nitrocellulose membranes using a Trans-Blot Turbo Transfer system (BIO-RAD) and probed with α-BIM (rabbit mAb (C34C5) and α-p21^WAF1/CIP1^ (rabbit mAb (12D1), (Cell signaling, Danvers, MA, USA). β-actin (mouse mAb (ab6276, AC-15), (Abcam, Cambridge, UK) was used as loading control. Blots were counterstained with IRDye 680/800-labeled secondary antibodies (LI-COR biosciences, Lincoln, NE, USA).

### Statistical analyses

The miRNAs differential expression analysis was performed using a Bayesian approach that has been implemented in the R-package ShrinkBayes. This approach has been known for its robustness in small sample series [38]. P-values < 0.05 were considered statistically significant for a single test, and Benjamini-Hochberg corrected false discovery rates (FDR) < 0.1 were considered statistically significant for multiple tests [39]. Statistical analyses were performed using R, version 3.0. MiRNA profiles were compared using global testing with a multinomal regression model [40].

Difference between mRNA and miRNA expression levels between paired initial diagnosis-relapse samples, as measured by Taqman, were determined using the one-sided Wilcoxon paired signed rank test.

## SUPPLEMENTARY TABLES AND FIGURES



## References

[1] Balgobind BV, Hollink IH, Arentsen-Peters ST, Zimmermann M, Harbott J, Beverloo HB, von Bergh AR, Cloos J, Kaspers GJ, de Haas V, Zemanova Z, Stary J, Cayuela JM (2011). Integrative analysis of type-I and type-II aberrations underscores the genetic heterogeneity of pediatric acute myeloid leukemia. Haematologica.

[2] Creutzig U, van den Heuvel-Eibrink MM, Gibson B, Dworzak MN, Adachi S, de Bont E, Harbott J, Hasle H, Johnston D, Kinoshita A, Lehrnbecher T, Leverger G, Mejstrikova E (2012). Diagnosis and management of acute myeloid leukemia in children and adolescents: recommendations from an international expert panel. Blood.

[3] Balgobind BV, Zwaan CM, Pieters R, Van den Heuvel-Eibrink MM (2011). The heterogeneity of pediatric MLL-rearranged acute myeloid leukemia. Leukemia.

[4] Balgobind BV, Raimondi SC, Harbott J, Zimmermann M, Alonzo TA, Auvrignon A, Beverloo HB, Chang M, Creutzig U, Dworzak MN, Forestier E, Gibson B, Hasle H (2009). Novel prognostic subgroups in childhood 11q23/MLL-rearranged acute myeloid leukemia: results of an international retrospective study. Blood.

[5] Fanini F, Vannini I, Fabbri M (2009). MicroRNAs: tiny players with a big role in the pathogenesis of leukemias and lymphomas. Hematology Reviews.

[6] Calin GA, Sevignani C, Dumitru CD, Hyslop T, Noch E, Yendamuri S, Shimizu M, Rattan S, Bullrich F, Negrini M, Croce CM (2004). Human microRNA genes are frequently located at fragile sites and genomic regions involved in cancers. Proc Natl Acad Sci U S A.

[7] Croce CM (2009). Causes and consequences of microRNA dysregulation in cancer. Nat Rev Genet.

[8] Esquela-Kerscher A, Slack FJ (2006). Oncomirs - microRNAs with a role in cancer. Nat Rev Cancer.

[9] Erhard F, Haas J, Lieber D, Malterer G, Jaskiewicz L, Zavolan M, Dölken L, Zimmer R (2014). Widespread context dependency of microRNAmediated regulation. Genome Research.

[10] Bonauer A, Dimmeler S (2009). The microRNA-17-92 cluster: still a miRacle?. Cell Cycle.

[11] Mi S, Li Z, Chen P, He C, Cao D, Elkahloun A, Lu J, Pelloso LA, Wunderlich M, Huang H, Luo RT, Sun M, He M (2010). Aberrant overexpression and function of the miR-17-92 cluster in MLL-rearranged acute leukemia. Proc Natl Acad Sci U S A.

[12] Saetrom P, Snove O, Rossi JJ (2007). Epigenetics and microRNAs. Pediatr Res.

[13] Emmrich S, Katsman-Kuipers JE, Henke K, Khatib ME, Jammal R, Engeland F, Dasci F, Zwaan CM, den Boer ML, Verboon L, Stary J, Baruchel A, de Haas V (2014). miR-9 is a tumor suppressor in pediatric AML with t(8;21). Leukemia.

[14] Kim YK, Kim VN (2007). Processing of intronic microRNAs. Embo J.

[15] Petrocca F, Vecchione A, Croce CM (2008). Emerging role of miR-106b-25/miR-17-92 clusters in the control of transforming growth factor beta signaling. Cancer Res.

[16] Pillaire MJ, Selves J, Gordien K, Gourraud PA, Gentil C, Danjoux M, Do C, Negre V, Bieth A, Guimbaud R, Trouche D, Pasero P, Mechali M (2010). A ‘DNA replication’ signature of progression and negative outcome in colorectal cancer. Oncogene.

[17] Agranat-Tamir L, Shomron N, Sperling J, Sperling R (2014). Interplay between pre-mRNA splicing and microRNA biogenesis within the supraspliceosome. Nucleic Acids Res.

[18] Petrocca F, Visone R, Onelli MR, Shah MH, Nicoloso MS, de Martino I, Iliopoulos D, Pilozzi E, Liu CG, Negrini M, Cavazzini L, Volinia S, Alder H (2008). E2F1-regulated microRNAs impair TGFbeta-dependent cell-cycle arrest and apoptosis in gastric cancer. Cancer Cell.

[19] Ivanovska I, Ball AS, Diaz RL, Magnus JF, Kibukawa M, Schelter JM, Kobayashi SV, Lim L, Burchard J, Jackson AL, Linsley PS, Cleary MA (2008). MicroRNAs in the miR-106b family regulate p21/CDKN1A and promote cell cycle progression. Mol Cell Biol.

[20] Calin GA, Croce CM (2006). MicroRNA signatures in human cancers. Nat Rev Cancer.

[21] Zhang H, Luo XQ, Zhang P, Huang LB, Zheng YS, Wu J, Zhou H, Qu LH, Xu L, Chen YQ (2009). MicroRNA patterns associated with clinical prognostic parameters and CNS relapse prediction in pediatric acute leukemia. PLoS One.

[22] Welch JS, Ley TJ, Link DC, Miller CA, Larson DE, Koboldt DC, Wartman LD, Lamprecht TL, Liu F, Xia J, Kandoth C, Fulton RS, McLellan MD (2012). The origin and evolution of mutations in acute myeloid leukemia. Cell.

[23] Ding L, Ley TJ, Larson DE, Miller CA, Koboldt DC, Welch JS, Ritchey JK, Young MA, Lamprecht T, McLellan MD, McMichael JF, Wallis JW, Lu C (2012). Clonal evolution in relapsed acute myeloid leukaemia revealed by whole-genome sequencing. Nature.

[24] Bachas C, Schuurhuis GJ, Zwaan CM, van den Heuvel-Eibrink MM, den Boer ML, de Bont ES, Kwidama ZJ, Reinhardt D, Creutzig U, de Haas V, Kaspers GJ, Cloos J (2015). Gene expression profiles associated with pediatric relapsed AML. PLoS One.

[25] Hudson RS, Yi M, Esposito D, Glynn SA, Starks AM, Yang Y, Schetter AJ, Watkins SK, Hurwitz AA, Dorsey TH, Stephens RM, Croce CM, Ambs S (2013). MicroRNA-106b-25 cluster expression is associated with early disease recurrence and targets caspase-7 and focal adhesion in human prostate cancer. Oncogene.

[26] Smith AL, Iwanaga R, Drasin DJ, Micalizzi DS, Vartuli RL, Tan AC, Ford HL (2012). The miR-106b-25 cluster targets Smad7, activates TGF-beta signaling, and induces EMT and tumor initiating cell characteristics downstream of Six1 in human breast cancer. Oncogene.

[27] Wang Y, Li Z, He C, Wang D, Yuan X, Chen J, Jin J (2010). MicroRNAs expression signatures are associated with lineage and survival in acute leukemias. Blood Cells Mol Dis.

[28] Zhi F, Cao X, Xie X, Wang B, Dong W, Gu W, Ling Y, Wang R, Yang Y, Liu Y (2013). Identification of circulating microRNAs as potential biomarkers for detecting acute myeloid leukemia. PLoS One.

[29] Blow JJ, Hodgson B (2002). Replication licensing--defining the proliferative state?. Trends Cell Biol.

[30] Dimova DK, Dyson NJ (2005). The E2F transcriptional network: old acquaintances with new faces. Oncogene.

[31] Tyagi S, Herr W (2009). E2F1 mediates DNA damage and apoptosis through HCF-1 and the MLL family of histone methyltransferases. Embo J.

[32] Ambs S, Prueitt RL, Yi M, Hudson RS, Howe TM, Petrocca F, Wallace TA, Liu CG, Volinia S, Calin GA, Yfantis HG, Stephens RM, Croce CM (2008). Genomic profiling of microRNA and messenger RNA reveals deregulated microRNA expression in prostate cancer. Cancer Res.

[33] Garofalo M, Romano G, Di Leva G, Nuovo G, Jeon YJ, Ngankeu A, Sun J, Lovat F, Alder H, Condorelli G, Engelman JA, Ono M, Rho JK (2012). EGFR and MET receptor tyrosine kinase-altered microRNA expression induces tumorigenesis and gefitinib resistance in lung cancers. Nat Med.

[34] Kaspers GJ, Veerman AJ, Pieters R, Broekema GJ, Huismans DR, Kazemier KM, Loonen AH, Rottier MA, van Zantwijk CH, Hahlen K (1994). Mononuclear cells contaminating acute lymphoblastic leukaemic samples tested for cellular drug resistance using the methyl-thiazol-tetrazolium assay. Br J Cancer.

[35] Van Vlierberghe P, van Grotel M, Beverloo HB, Lee C, Helgason T, Buijs-Gladdines J, Passier M, van Wering ER, Veerman AJ, Kamps WA, Meijerink JP, Pieters R (2006). The cryptic chromosomal deletion del(11)(p12p13) as a new activation mechanism of LMO2 in pediatric T-cell acute lymphoblastic leukemia. Blood.

[36] Stam RW, den Boer ML, Meijerink JP, Ebus ME, Peters GJ, Noordhuis P, Janka-Schaub GE, Armstrong SA, Korsmeyer SJ, Pieters R (2003). Differential mRNA expression of Ara-C-metabolizing enzymes explains Ara-C sensitivity in MLL gene-rearranged infant acute lymphoblastic leukemia. Blood.

[37] Balgobind BV, Zwaan CM, Reinhardt D, Arentsen-Peters TJ, Hollink IH, de Haas V, Kaspers GJ, de Bont ES, Baruchel A, Stary J, Meyer C, Marschalek R, Creutzig U (2010). High BRE expression in pediatric MLL-rearranged AML is associated with favorable outcome. Leukemia.

[38] Van De Wiel MA, Leday GG, Pardo L, Rue H, Van Der Vaart AW, Van Wieringen WN (2013). Bayesian analysis of RNA sequencing data by estimating multiple shrinkage priors. Biostatistics.

[39] Benjamini Y, Hochberg Y (1995). Controlling the False Discovery Rate: A Practical and Powerful Approach to Multiple Testing. Journal of the Royal Statistical Society Series B (Methodological).

[40] Goeman JJ, van de Geer SA, de Kort F, van Houwelingen HC (2004). A global test for groups of genes: testing association with a clinical outcome. Bioinformatics.

