# Supplementary material for: MicroRNA-106b~25 cluster is upregulated in relapsed MLL-rearranged pediatric acute myeloid leukemia
Source: Oncotarget. 2016 Jun 24;7(30):48412–22. doi: 10.18632/oncotarget.10270 (PMC5217027; doi:10.18632/oncotarget.10270)
Supplement: Supplementary file 1 [file oncotarget-07-48412-s001.pdf]

MicroRNA-106b~25 cluster is upregulated in relapsed *MLL*-rearranged pediatric acute myeloid leukemia

SUPPLEMENTARY TABLES AND FIGURES

Supplementary Table S1: Patient characteristics

		**Total n=127**
Sex	N	(%)
Male	72	(56.7)
Female	55	(43.3)
FAB		
M0	6	(5)
M1	15	(12)
M2	32	(25)
M3	8	(6)
M4	35	(28)
M5	22	(17)
M6	2	(2)
M7	3	(2)
Unknown	4	(3)
Cytogenetics		
MLL	23	(18)
t(8;21)	20	(16)
inv(16)	19	(15)
t(15;17)	7	(5)
t(7;12)	2	(2)
CN	28	(22)
Other/unknown	28	(22)
	**Median**	**Range**
Age (years)	9.2	(0.0-18.4)
WBC (x 10^9/L)	72.1	(1.4-475)

Supplementary Table S2: Patient characteristics *MLL*-rearranged patients

		**Total n= 23**
Sex	N	(%)
Male	15	(65.2)
Female	8	(34.8)
FAB		
M0	0	(0)
M1	1	(4)
M2	0	(0)
M3	0	(0)
M4	7	(31)
M5	14	(61)
M6	0	(0)
M7	0	(0)
Unknown	1	(4)
	**Median**	**Range**
Age (years)	5.3	(0.3-16.9)
WBC (x 10^9/L)	106.1	(2.3-475)

Supplementary Table S3: Average expression of validated miRNAs relative to RNU24, determined by stem loop RT-qPCR

miRNA	Average expression Diagnosis (range)	Average expression Relapse (range)	p-value
miR-30c	-0.5729 (-1.85– 0.29)	-0.0443 (-0.63– 1.06)	*0.0012*
miR-15b	-0.3364 (-1.79– 1.45)	0.3614 (-0.9 – 2.23)	*0.0026*
miR-18a	-4.365 (-5.53- -3.31)	-3.765 (-5.28- -2.86)	*0.0034*
miR-106b	-0.5729 (-2.24- 0.61)	-0.0443 (-1.36- 0.85)	*0.0164*
miR-500a	-6.3079 (-8.16- -4.89)	-5.6164 (-6.94- -3.41)	*0.0176*
miR-142-5p	-1.805 (-2.28- -0.39)	-1.1838 (-2.24- -0.76)	*0.0195*
miR-660	-3.9427 (-5.31- -2.65)	-3.3371 (-5.12- -1.35)	*0.0247*
miR-93	0.7686 (-0.5- 1.63)	1.1964 (-0.29- 2.36)	*0.0319*
miR-103	-1.4986 (-3.43- 0.07)	-1.1624 (-2.54- 0.09)	*0.0338*
miR-25	-0.7357 (-3.42- 0.3)	-0.2574 (-1.5- 0.79)	*0.0338*
miR-32	-6.3443 (-10.79- -3.92)	-5.645 (-7.97- 3.73)	*0.0392*
let-7a	0.5275 (-1.09- 4.83)	0.5225 (-0.37- 1.61)	*0.0398*
miR-362-3p	-9.8825 (-25- -6.64)	-7.0275 (-8.85- -6.21)	0.0547
miR-142-3p	2.4975 (-0.32- 4.07)	3.3375 (2.28- 3.67)	0.0742
miR-185	-2.9893 (-4.43- -0.73)	-2.7814 (-4.4- -0.86)	0.1338
miR-181a	-1.8257 (-4.43- 1.01)	-1.5993 (-4.23- 0.49)	0.1897
miR-532-5p	-4.6563 (-6.63- -2.31)	-4.1863 (-4.56- 3.63)	0.2203
miR-330-3p	-10.5663 (-11.09- -9.7)	-10.3900 (-11.12- -9.09)	0.2704
miR-423-5p	-3.8863 (--5.71- -0.56)	-3.7963 (-4.79- -2.63)	0.3203
miR-181a-2-3p	-5.1171 (-6.73- -5.53)	-4.9179 (-6.62- -3.86)	0.3349
miR-181c	-5.7614 (-10.72- 0.3)	-5.7386 (-9.98- -0.95)	0.3349
miR-532	ND	ND	

P-values were determined using the one sided Wilcoxon paired signed rank test, significant P-values (<0.05) in italics. ND; no expression detectable.

Average miRNA expression of validated miRNAs in diagnosis and relapse samples of paired samples with *MLL*-rearrangements.

Supplementary Table S4: Overview of primer sequences for mRNA qRT-PCR of E2F1, MCM7, p21, and BIM

P21	Fw	5’-CAC-CCA-CGA-ATG-GTT-ATC-3’
	Rv	5’-CGC-TGG-CTG-CAT-AAT-AAT-3’
BIM	Fw	5’-AAG-GCC-CGC-TCT-ACA-T-3’
	Rv	5’-TCC-GGG-AGA-GAG-GAA-A-3’
E2F1	Fw	5’-GGC-CTG-GGT-GAT-TTA-TTT-3’
	Rv	5’-GGC-GCT-TCA-GAC-ACT-G-3’
MCM7	Fw	5’-GGG-CAG-ACA-GCT-AGG-ACT-3’
	Rv	5’-CAG-GCT-GGA-ATC-AGA-CAA-3’

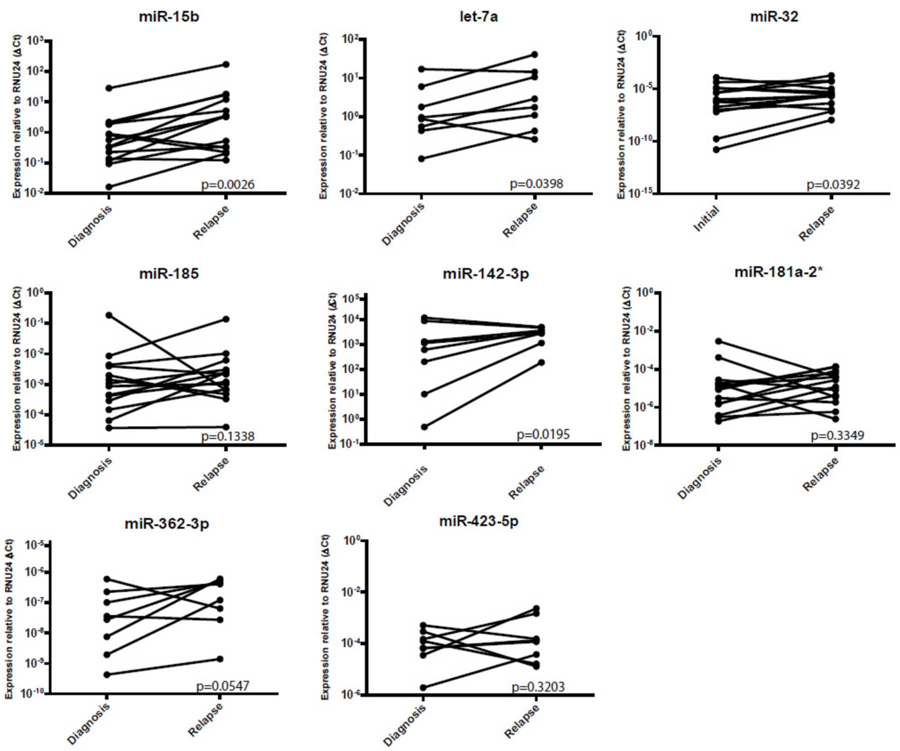

Supplementary Figure S1: Validation of top 10 differentially expressed miRNAs in paired pediatric AML intial diagnosis-relapse cases with stem-loop RT-qPCR. MiR-15b, let-7a, miR-32, and miR-142-3p are significantly overexpressed in relapse ($p<0.05$). MiR-532-3p is not shown due to undetectable miRNAs expression. Data are presented with P-values one sided.

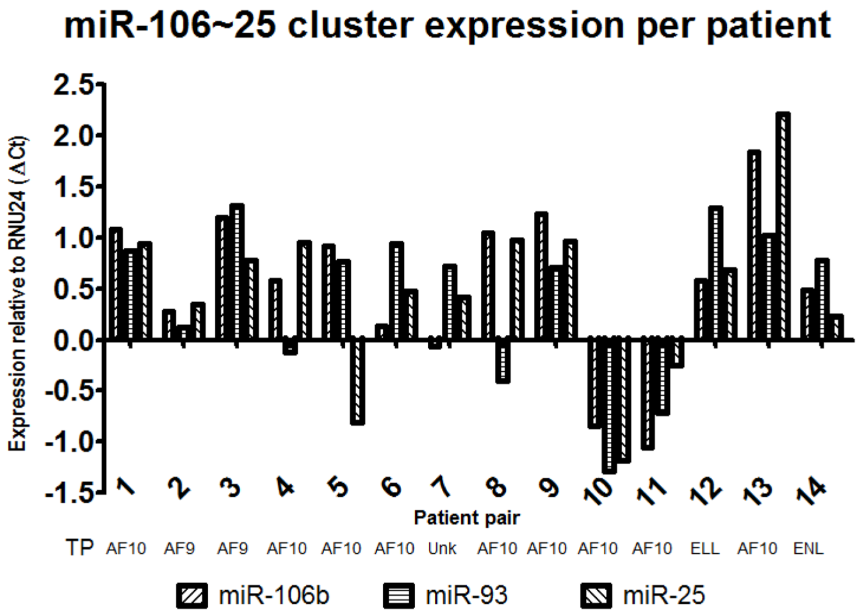

Supplementary Figure S2: Cluster expression in paired pediatric AML initial diagnosis- relapse cases. Relative expression of miR-25, miR-93, and miR-106b per patient. Four patient have one miRNA that is expressed in the opposite direction. Translocation partners (TPs) are depicted under each patient. Unk: unknown.
